# The Dilemmas of Leading Health Organizations in Complex Settings: Comment on "Dual Agency in Hospitals: What Strategies Do Managers and Physicians Apply to Reconcile Dilemmas Between Clinical and Economic Considerations?"

**DOI:** 10.34172/ijhpm.2022.7252

**Published:** 2022-08-15

**Authors:** Ian Greener

**Affiliations:** University of Strathclyde, Glasgow, UK.

**Keywords:** Dilemmas, Dual-Agency, Decision-Making

## Abstract

Waitzberg and colleagues’ research explores hospital managers, chief physicians and other physicians in German and Israeli hospitals, making use of thematic analysis to explore what they call ‘dilemmas’ between the commitments to clinical needs, and their hospitals’ financial sustainability. This commentary will provide a summary of the paper, into which I will embed some items I will follow-up on in my second half. The second half will then explore these items in greater depth, considering the strengths and weaknesses of the article. I then make some suggestions for future work based around the findings the authors present in terms of managerial and clinical identity, how compromises are reached in hospital settings, and how we compare different health systems.

 In their article, Waitzberg et al^1^ show how hospitals have multiple objectives which have the possibility of falling out of alignment with one another, and may even threaten institutional survival. However, in institutions of any complexity, where professional groups have appeared which view key performance measures through different lenses, such misalignment is likely to be common.

 Equally, all hospital professionals are located in complex relationships of one kind or another. They have their own professional identities, but also face demands from other groups which put in place control systems to try and get the professionals to change their behaviours. Governments are clearly significant stakeholders in healthcare provision, paying (at least indirectly) for a large proportion of it (even in a country with a large private health sector such as Israel), and will have a view of how healthcare systems should change. In more privatised settings shareholders will be expecting returns on their investments. If professionals are salaried and consider their employment to be secure, this may mean cost-based considerations may not be most important for them. The standard critique of managers in this situation is that they will focus on maximising their own welfare (bigger offices, higher pay) whereas doctors may focus on individual patient need and not see that expensive treatments may jeopardise the ability to treat other groups who are also in need, but may also seek greater autonomy from managerial control and higher pay too.

 Germany and Israel represent interesting countries to compare in these terms, both having statutory health insurance redistributed to non-profit insurers which purchase hospital services through managed competition systems. Both systems link hospital payment to activity, linked in turn to the coding of treatments, albeit with slightly different systems. However, as the authors point out, the demography of Germany is somewhat different to that of Israel, leading to the system facing different kinds of health challenges.

 The interviews were analysed using thematic analysis, with a large amount of cross-checking and coding between the research team, and with the themes being derived from both existing research and more inductively from the interviews themselves.

 The results describe three archetypal situations. The first is where activity-based payment systems were in alignment with clinical assessments. In these situations, there was scope for greater efficiency to be achieved by looking carefully at clinical practices, perhaps reducing length of stay or removing unnecessary expenditures, for example. This might involve changes to clinical practice, but allowed greater number of patients to be treated, and so, under payment systems linked to activity, more resources for the hospital that could be framed as a ‘win-win’ for both doctors and managers.

 However, there were also situations where clinical practice and financial incentive systems are misaligned, and the researchers found that, in these situations, this required hospital professionals to engage in a range of practices to ‘reshape management’ to try and balance this misalignment through strategies such as planning treatment courses more strategically, or perhaps by recoding the treatments to ensure greater revenues. In either case, the treatments themselves were not significantly changed – this was about maximising the revenue per case.

 The third theme was ‘reframing decision-making’ to try and structure decision making in ways that brought clinical and managerial practices more into alignment again. This could be done by trying to refocus away from individual patients to more collective ‘average’ counting systems to try and make sure revenue targets were reached, or by trying to agree decision-making criteria in advance, perhaps through the use of toolkits, which then constrained both doctors and managers, but on an agreed basis.

 All three themes put in place fragile agreements, and required active co-operation between different professional groups to prevent them from failing. However, the message of the article is hopeful in suggesting that they present ideas for how conflicts can be overcome to enable both clinical and managerial objectives to be met.

 It is also important to note that identifying groups as unequivocally doctors or managers may not reflect the complex reality faced by hospital professionals – who often occupy professional hybrid roles which involve them having to balance the needs of both groups. Equally, it is easy to dichotomise interests into ‘managerial’ and ‘clinical’ when most professionals in either grouping, even if they are not in explicitly hybrid roles, will be concerned with the other group’s priorities. Waitzberg et al show that this analysis extends to Chief Executives and Chief Financial Officers who showed an ability to understand clinical considerations in their decision-making. Making this clear adds valuable nuance to the discussion.

 There were also differences between the two health systems. Germany’s better-resourced system which has a larger number of smaller providers made it more possible for specialization to occur than in Israel, and for patients to be more likely to be moved to other care settings. The lack of these factors in Israel meant that bottlenecks in discharging patients were more likely to occur there, but at the same time seemed to make Israeli doctors more sympathetic to arguments from managers to improve efficiency.

 Waitzberg et al also show two possible weaknesses to the study – a possible bias towards the successful stories about co-operation being reported because of the focus in the research of ‘what works,’ and of there being other kinds of dilemmas beyond those that misalign economic and clinical considerations – which they acknowledge but promise to pick up in future work. I will say more on this below.

 The paper has significant strengths. Outlining the complexities of trying to balance clinical and managerial needs is important work, and being able to offer examples where compromises have been found that can be ‘win-win’ is a valuable contribution. Too often doctors and managers are still presented as being inextricably in opposition to one another, when this does not always need to be the case.

## Identity, Compromise and Comparison

 It is also really valuable to talk about overlaps between clinical and managerial roles, and the issues of identity that these raise. I would have liked a little more on this though. Is it still the case, as earlier work suggested^2^ that doctors tend to occupy managerial roles for a defined period only, and then return to their professional practice, and so tend to be restrained in what they do as managers, aware that they have to return to their professional peers? Are there doctors which consciously decide to leave practice behind, and how did they come to that decision? Are doctors which take on managerial roles harder on their professional colleagues or better able to find compromises? There are still huge research opportunities in this space, and I hope the team will have the opportunity to dig a little more into their current data, or conduct new research, to help us understand the nuances of this area even further.

 Another aspect of the paper I really liked was its emphasis on the fragility of the compromises that were reached between clinical and financial goals. This makes clear the importance of showing these compromises are areas which have to be continually worked on, rather than just taken-for-granted. This highlights the importance of trying to avoid simple answers to complex problems. Health services research can be focussed on ‘what works’ when the answer to that question is often much more complex,^3^ and will depend on the histories of the decision-makers who are trying to find answers, their degree of trust, and the issue in hand. Adding that nuance is really valuable.

 An area which is perhaps a little over-looked in the paper, and which the authors are clear is a limitation, is in identifying situations in which agreements and compromises could not be reached. This is an understandable omission given the focus of the research, and the allowed word limits for the paper. However, it would have been good to see what failure situations could tell us in terms of situations which managers and doctors might seek to avoid, and how they might try and avoid them appearing. Failure is an area where there is now fascinating organizational research,^4^ and I think the authors could make an important contribution in considering this area in relation to health services.

 A final area which I would have liked to see more was in terms of cross-country comparison. There were two countries in the study which are very different. Although the paper is clear about the commonalties of dilemma they found in their research, I would also have liked to understand more about the differences that the healthcare settings made. Qualitative research is often afraid to generalise beyond individual sites, and I am pleased that this paper did not fall into the trap of wanting to regard every case study as distinct, but looking at how the healthcare setting context influenced the findings in greater depth would have added an additional layer.^5^ What is in the paper is interesting, but I found myself wanting to read more.

 In all, this is a really valuable paper. It brings issues raised in previous research (but which are often still overlooked) back into focus, and its positive emphasis on how compromises can be found is extremely welcome at a time when too often the challenges of working in complex healthcare settings are portrayed as being insurmountable.

## Ethical issues

 Not applicable.

## Competing interests

 Author declares that he has no competing interests.

## Author’s contribution

 IG is the single author of the paper.
